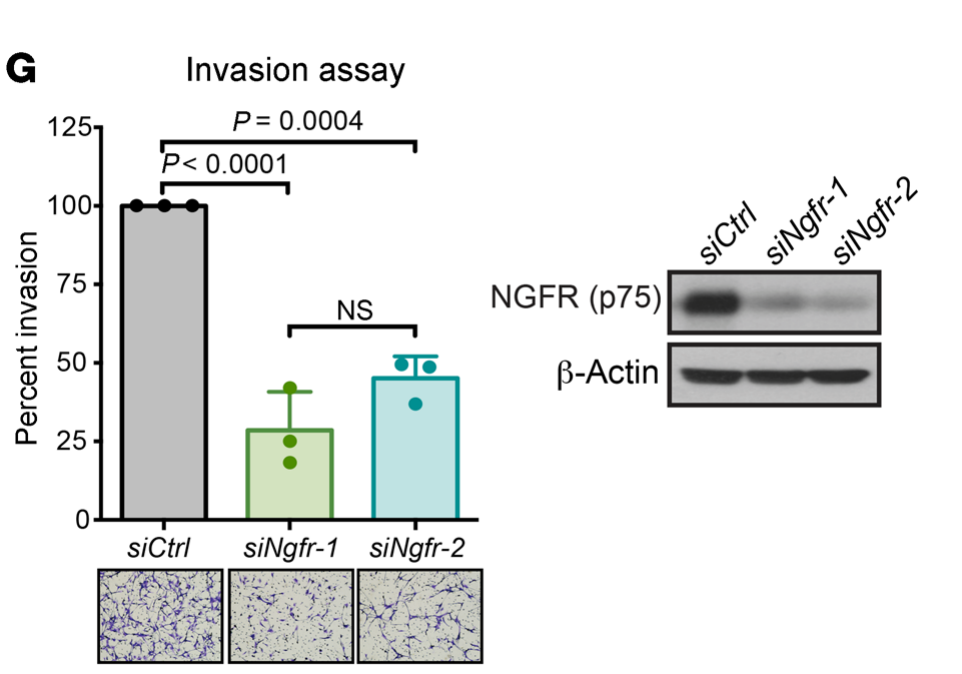# Inhibiting the MNK1/2-eIF4E axis impairs melanoma phenotype switching and potentiates antitumor immune responses

**DOI:** 10.1172/JCI181575

**Published:** 2024-05-01

**Authors:** Fan Huang, Christophe Gonçalves, Margarita Bartish, Joelle Rémy-Sarrazin, Mark E. Issa, Brendan Cordeiro, Qianyu Guo, Audrey Emond, Mikhael Attias, William Yang, Dany Plourde, Jie Su, Marina Godoy Gimeno, Yao Zhan, Alba Galán, Tomasz Rzymski, Milena Mazan, Magdalena Masiejczyk, Jacek Faber, Elie Khoury, Alexandre Benoit, Natascha Gagnon, David Dankort, Fabrice Journe, Ghanem E. Ghanem, Connie M. Krawczyk, H. Uri Saragovi, Ciriaco A. Piccirillo, Nahum Sonenberg, Ivan Topisirovic, Christopher E. Rudd, Wilson H. Miller, Sonia V. del Rincón

Original citation: *J Clin Invest*. 2021;131(8):e140752. https://doi.org/10.1172/JCI140752

Citation for this corrigendum: *J Clin Invest*. 2024;134(9):e181575. https://doi.org/10.1172/JCI181575

The authors recently became aware that in the legend for Figure 1D, the sample number was incorrect. In addition, in Figure 3G, the loading control was incorrectly labelled. Finally, there were errors in the figure legends for the supplemental figures. The correct legend for Figure 1D and figure panel for Figure 3G appear below. The HTML and PDF versions of the article and supplemental material have been updated online.

(**D**) Percentages (left) and representative images (right) of Ki67-positive melanoma cells in eIF4E^WT^ and eIF4E^KI^ primary melanoma sections (day 50; *n* = 8 per genotype; scale bars: 50 μm).

The authors regret the errors.

## Figures and Tables

**Figure:**